# A simple method to measure sulfonation in man using paracetamol as probe drug

**DOI:** 10.1038/s41598-021-88393-3

**Published:** 2021-04-27

**Authors:** Natália Marto, Judit Morello, Alexandra M. M. Antunes, Sofia Azeredo, Emília C. Monteiro, Sofia A. Pereira

**Affiliations:** 1 NOVA Medical School, iNOVA4Health, CEDOC-Chronic Diseases Research Center, Edifício CEDOC II, Universidade NOVA de Lisboa, Rua Câmara Pestana no. 6, lab 3.16, 1150-082 Lisboa, Portugal; 2 Department of Internal Medicine, Hospital da Luz Lisboa, Avenida Lusíada, 100, 1500-650 Lisboa, Portugal; 3 Instituto Superior Técnico, ULisboa, Centro de Química Estrutural, Av. Rovisco Pais, 1049-001 Lisboa, Portugal; 4 NOVA Medical School, Universidade NOVA de Lisboa, Campo Mártires da Pátria, 130, 1169-056 Lisboa, Portugal

**Keywords:** Metabolomics, Drug safety, Transferases, Clinical pharmacology

## Abstract

Sulfotransferase enzymes (SULT) catalyse sulfoconjugation of drugs, as well as endogenous mediators, gut microbiota metabolites and environmental xenobiotics. To address the limited evidence on sulfonation activity from clinical research, we developed a clinical metabolic phenotyping method using paracetamol as a probe substrate. Our aim was to estimate sulfonation capability of phenolic compounds and study its intraindividual variability in man. A total of 36 healthy adult volunteers (12 men, 12 women and 12 women on oral contraceptives) received paracetamol in a 1 g-tablet formulation on three separate occasions. Paracetamol and its metabolites were measured in plasma and spot urine samples using liquid chromatography-high resolution mass spectrometry. A metabolic ratio (Paracetamol Sulfonation Index—PSI) was used to estimate phenol SULT activity. PSI showed low intraindividual variability, with a good correlation between values in plasma and spot urine samples. Urinary PSI was independent of factors not related to SULT activity, such as urine pH or eGFR. Gender and oral contraceptive intake had no impact on PSI. Our SULT phenotyping method is a simple non-invasive procedure requiring urine spot samples, using the safe and convenient drug paracetamol as a probe substrate, and with low intraindividual coefficient of variation. Although it will not give us mechanistic information, it will provide us an empirical measure of an individual’s sulfonator status. To the best of our knowledge, our method provides the first standardised in vivo empirical measure of an individual’s phenol sulfonation capability and of its intraindividual variability. EUDRA-CT 2016-001395-29, NCT03182595 June 9, 2017.

## Introduction

The cytosolic sulfotransferase enzymes (SULT) are products of a diverse gene superfamily that catalyse sulfoconjugation (or sulfonation)—a relatively understudied topic in the area of drug metabolism—using 3′-phosphoadenosine 5′-phosphosulfate (PAPS) as a sulfate donor. First-pass effect organs, such as the liver and the small intestine, contain the largest overall amount of SULT, while the kidney and lung contain low levels of SULT^1^. Besides involvement in drug metabolism, SULT are at the crossroad of metabolic pathways of endogenous compounds (e.g. estradiol, thyroid hormones, catecholamines), diverse environmental xenobiotics and even human-gut microbiome metabolites (e.g. cresols and indoles), all with the potential to compete for sulfonation and reciprocally influence biotransformation^2,3^.

Among SULT involved in xenobiotic and drug biotransformation, SULT1A1 is particularly relevant, due to its broadest substrate specificity and extensive tissue distribution, being the major enzyme in the liver^2^. SULT1A1 catalyses with high affinity sulfonation of many phenolic molecules, including estradiol and thyroid hormones, several environmental mutagens and carcinogens, gut microbiome metabolites, and drugs (e.g. paracetamol, ethinylestradiol, levodopa, opioid drugs, propofol, tedizolid, fulvestrant, and tamoxifen’s active metabolites)^4–7^.

Another SULT important in drug metabolism is SULT1A3, which is specific to primates and catalyses the sulfonation of catecholamines and drugs such as salbutamol, paracetamol, morphine, tramadol metabolites, tapentadol, levodopa, and troglitazone^2,8–11^. In adults, SULT1A3 is a major extrahepatic enzyme, particularly abundant in the small intestine, with implications for the oral bioavailability of a number of drugs and dietary compounds that are its substrates^12^.

Race-related genetic variation in SULT enzymes includes single nucleotide polymorphisms and copy number variation, which have been shown to significantly determine enzyme activity^13,14^. Nevertheless, genetic variation only explains a minor part of interindividual variability, suggesting regulation by nuclear receptors, PAPS-cofactor availability and inhibition by various drugs and environmental compounds could be major determinants of variability in SULT activity^15^. This variability in sulfonation capacity may determine therapeutic failure or toxicity of drugs metabolized by SULT, underlie drug interactions and interfere with the metabolism of environmental and dietary chemicals or endogenous compounds, with possible implications in disease and uncharted adverse drug reactions^15^.

In contrast to phase I enzymes, there is limited evidence on sulfonation activity from clinical research, although in vivo studies are of key importance in assessing the functional consequences of individual variation^16^. Current knowledge on SULT interindividual variability is mostly based on animal studies and on in vitro pharmacogenetic and expression/activity studies^15^. These data are hampered by relevant interspecies differences in SULT expression and the technical difficulties in maintaining the activity of cytosolic enzymes in vitro^2,15^. Clinical evidence on SULT activity derives mostly from Rosemary Waring’s clinical studies on sulphur biotransformation pathways in specific chronic diseases, which introduced paracetamol as a probe drug to estimate sulfation capacity^17–22^. Occasional epidemiological studies have focused on the associations between SULT genotype and cancer risk and treatment response^23^.

In implementing precision medicine initiatives for SULT-mediated drug metabolism, we must identify methods that will likely produce accurate evidence of enzyme activities (phenotypes). As part of our group's focus on precision medicine^24,25^, particularly on the uniqueness of the individual’s metabolic capability and its implications for drug response, we strived to develop a simple method for estimating sulfonation capacity in the clinical setting.

Metabolic phenotyping is a well-established field involving the analysis of metabolites in body fluids using various spectroscopic methods to provide information on the metabolic phenotype of individuals or populations^26,27^. The metabolic phenotype is the characteristic metabolite profile reflecting the host genome and its interaction with environmental factors, diet, and the gut microbiome, and can be used to analyse the relationships between genetic variations and environmental triggers of disease and to study drug response^26,28^. Current research is using metabolic phenotype to describe real-time enzyme activity through the calculation of a product-to-substrate ratio for a particular enzyme of interest^29^. In our case, we are calculating a sulfonated-metabolite to probe substrate ratio.

**Figure 1:**
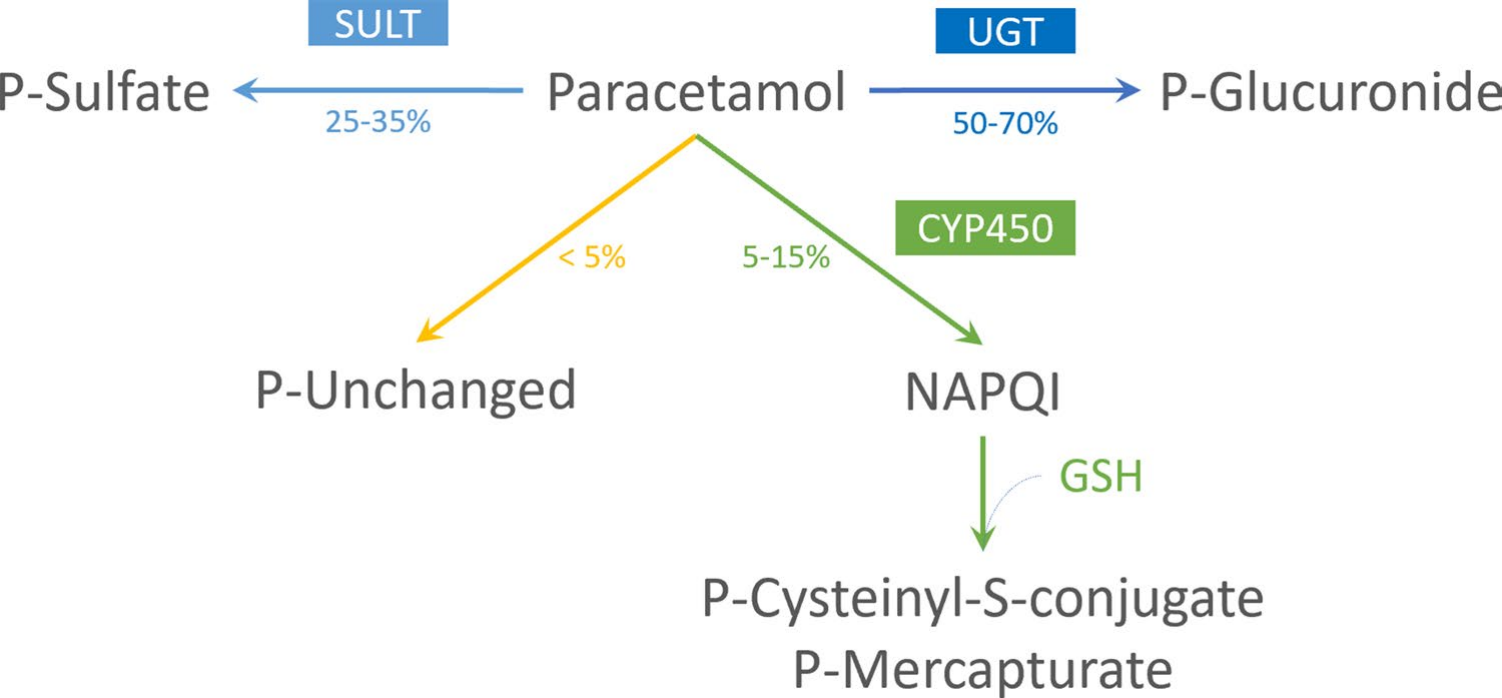
Paracetamol metabolism overview. *P* paracetamol, *SULT* sulfotransferase, *UGT* uridine 5′-diphosphoglucuronosyltransferase, *CYP450* cytochrome P450, *GSH* glutathione, *NAPQI* *N*-acetyl-*p*-benzoquinone imine.

Echoing RH Waring’s pioneering work, our clinical metabolic phenotyping method uses paracetamol as a probe substrate. Paracetamol is mostly metabolized by phase II reactions of biotransformation and its sulfonation accounts for up to 35% of the total paracetamol metabolism in humans. (Fig. 1) In vitro studies have shown paracetamol sulfonation to be mediated by several SULT isoforms, including SULT1A1, SULT1A3, SULT1C4, SULT1E1 and SULT2A1^10,30^. Nevertheless, studies in human organ samples have shown that sulfonation of paracetamol occurs mainly in liver and small intestine, where SULT1A1 and SULT1A3 are particularly abundant, respectively^10^. Although it has the highest affinity for paracetamol, SULT1C4 is primarily expressed in the foetal liver and in small quantities in lung and kidney, assuming a minor role in paracetamol metabolism in postnatal life^10,12^. While SULT1E1 and SULT2A1 are capable of sulfonating paracetamol at high concentrations, these isoforms probably have irrelevant activity in vivo^10^. Hence, paracetamol sulfonation reflects mainly the activity of SULT1A1 and 1A3 and could be used as an estimate of the activity of these enzymes, to stratify patients according to their phenol sulfonation capability.

Here, we report the results of our pilot clinical study testing a simple experimental protocol and analytical method using a metabolite ratio after ingestion of paracetamol for assessment of phenol sulfonation phenotype and analysis of intraindividual variability in healthy volunteers. To the best of our knowledge, so far there is no data on intraindividual variability for SULT activity in man.

## Materials and methods

### Ethics

The following protocol is approved by the Portuguese National Ethics Committee (CEIC code 20160561) and the Institutional Review Board at Hospital da Luz, SA, Lisbon, Portugal (IRB protocol HL_001_2016). The study conforms to Declaration of Helsinki, European Medicines Agency Guidelines for Good Clinical Practice and local regulations and is registered at the European Union Drug Regulating Authorities Clinical Trials Database (EUDRA-CT 2016-001395-29) and at ClinicalTrials.gov (NCT03182595). Written informed consent was obtained from all subjects.

### Study population

Assuming SULT activity might vary between 0 and 60% and estimating standard deviation (SD) as SD = range/4^31^, with a margin of error of 5% (E = 0.05) and a confidence level of 95% (z = 1.96), we calculated a sample size of n = 35 (> 34,57). To allow for stratification by sex and by oral contraceptive use, we recruited 12 men and 24 women (12 on oral contraceptives).

We screened adult volunteers from the community: a thorough history was obtained from all the subjects and each subject had a physical examination, blood tests and, for women, a urine pregnancy test. Patients were excluded if they reported significant disease, were on regular medication or had intake of any medication within 14 days before the beginning of the study (except for oral contraceptives). Subjects with a history of allergy or any contraindication to paracetamol were also excluded. Eligible subjects had screening blood tests within normal range and a negative pregnancy test.

### Study design

Eligible subjects had baseline blood and urine sampling (visit 1) and a further run-in period that included three sampling moments (visits 2 through 4), once every four weeks. At each of these three visits, subjects received paracetamol in a 1 g-tablet formulation (Ben-U-Ron, *Bene Farmacêutica Lda*., Lisbon, Portugal), to be taken orally with water, and had blood and urine samples collected 2 h after paracetamol intake; this time point was selected according to plasma concentration–time curves for paracetamol and its metabolites derived from published pharmacokinetic studies^32,33^. Aliquots were taken and stored at − 80 °C until analysis.

### Analytical procedures

The relative levels of paracetamol and its metabolites (glucuronide, sulfate, cysteinyl-S-conjugate and mercapturate) were assessed in plasma and urine using liquid chromatography-high resolution mass spectrometry (LC-HRMS). The pH was measured for all urine samples. Glomerular filtration rate (eGFR) was estimated for every subject using the CKD-EPI Creatinine 2009 Equation^34^.

#### Urine and plasma sample treatment and liquid chromatography-high resolution mass spectrometry (LC-HRMS)

Urine and plasma samples were analysed separately, using the same methodology. Samples were randomized before extraction.

A volume of 10 µL of each sample were pooled (quality control pool) and processed together with the samples to check the performance of the sample treatment and the LC-HRMS acquisition. Samples were processed upon a protein precipitation protocol^35^. Briefly, 150 µL of cold ethanol were mixed with 50 µL of sample. Samples were vortexed and placed at − 20 °C for 20 min. Then, samples were centrifuged at 3660 *g* for 10 min and 150 µL of the supernatant were transferred to a new microcentrifuge tube for dry vacuum. Dried samples were reconstituted with 10% acetonitrile in water: 400 µL in the case of urine or 150 µL in the case of plasma.

Samples were analysed by ultra-high-performance liquid chromatography (Elute UHPLC, Bruker, Bremen, Germany) interfaced with a Bruker Impact II quadrupole time-of-flight mass spectrometer equipped with an electrospray source (Bruker, Bremen, Germany). Chromatographic separation was performed on a CORTECS T3 column (1.6 µm, 2.1 × 100 mm) protected with a CORTECS T3 VanGuard pre-column (1.6 µm, 2.1 × 5 mm). The temperature of the column was set to 45 °C. The mobile phases consisted of water with 0.1% v/v of formic acid (phase A) and acetonitrile with 0.1% v/v of formic acid (phase B). The gradient was as follows: 0 min 100% phase A, then in 1 min 95% phase A, in 5 min 50% phase A, in 4 min 0% phase A, held for 8 min at 0% phase A, and then in 1 min to 100% phase A, held for 6 min at 100% phase A. The injection volume was 3 µL and the flow rate was 400 µL/min.

The high-resolution mass spectra were acquired in ESI positive mode, with the optimized parameters set as follows: ion spray voltage, + 4.5 kV; end plate offset, 500 V; nebulizer gas (N_2_), 4 bars; dry gas (N_2_), 8 L min^−1^; dry heater 200 °C. Internal calibration was performed on the high-precision calibration mode (HPC) with a solution of sodium formate 10 mM introduced to the ion source via a 20 µL loop at the beginning of each analysis, using a six-port valve. Acquisition was performed in full scan mode in the m/z 50–1000 range with a spectra rate of 1 Hz.

For compound identification, pseudo-multiple reaction monitoring (pseudoMRM) experiments were performed with the calculated exact m/z values of paracetamol and paracetamol metabolites (see Supplementary Tables S1 and S2 online). Besides, a paracetamol standard solution was injected to check the retention time of paracetamol.

#### Targeted peak detection of paracetamol and paracetamol metabolites

Paracetamol and paracetamol metabolites were identified from the expected m/z values of the precursor and product ions of paracetamol and paracetamol metabolites previously described^35,36^. Both measured accurate m/z and mSigma values were annotated for each ion formula (see Supplementary Tables S1 and S2 online). Paracetamol retention time was also verified with a paracetamol standard solution.

The acquired LC–MS data files from visits V2, V3 and V4 (98 samples) were converted to *.mzXML files using the ProteWizard MSconvert tool^37^. A targeted analysis was then performed with the open-source software MZmine^38,39^ and consisted of target peak detection, correction of retention time and peak matching.

Targeted peak detection was performed with the list of the corresponding m/z and retention time values for each precursor compound (see Supplementary Table S1 online) and the following parameters: shape tolerance = 10%, noise level = 1000, m/z tolerance = 0.005 Da or 20 ppm and retention time tolerance = 0.25 – 0.50 min (depending on the sample due to a retention time shift). Peak matching among samples was performed using the Join aligner method with m/z tolerance = 0.005 Da or 20 ppm, retention time tolerance = 0.5 (plasma samples) or 0.7 (urine samples) minute, weight for m/z and retention time = 1 and require same identification.

### Phenotype assignment

Paracetamol sulfonation index (PSI) was used to express phenol SULT activity. Three ratios were evaluated as candidates for PSI, as follows: PS/P, PS/(P + PS + PG) and PS/(P + PS + PG + PM + PC). All ratios were calculated for plasma and urine samples.

### Data and statistical analysis

Before statistical analysis, data were normalized by the total area of the chromatographic peaks corresponding to paracetamol and the metabolites of paracetamol. Descriptive statistics are presented as means and standard deviation. A P value less than 0.01 was considered to be statistically significant.

The demographic, background and baseline data are presented descriptively.

A frequency distribution analysis was performed for PSI. We calculated the intraindividual coefficient of variation (CV) expressed in percentage %. Correlation analysis was performed by calculating Pearson’s coefficient of correlation. The Kruskal–Wallis test was used to detect differences in PSI between groups.

All statistical analyses were performed using GraphPad Prism 7.0 software.

### Ethics approval

The protocol is approved by the Portuguese National Ethics Committee (CEIC code 20160561) and the Institutional Review Board at Hospital da Luz, SA, Lisbon, Portugal (IRB protocol HL_001_2016). The study conforms to Declaration of Helsinki, European Medicines Agency Guidelines for Good Clinical Practice and local regulations and is registered at the European Union Drug Regulating Authorities Clinical Trials Database (EUDRA-CT 2016–001,395-29) and at ClinicalTrials.gov (NCT03182595).

### Consent to participate

Written informed consent was obtained from all subjects.

## Results

Thirty-six adults were enrolled in our study, including 12 men, 12 women on oral contraceptives and 12 women off oral contraceptives. ﻿ All subjects were self-reported as healthy and used no medication. Table 1 summarizes the clinical characteristics of the 36 included subjects. There were no significant differences between the three groups. All subjects completed phenotyping visits. Two subjects missed the appointments for two of the phenotyping visits.

**Table 1:** Characteristics of the study population.

Variable	All subjects n = 36	Men n = 12	Women n = 24
Age (years) (mean and standard deviation)	34 (11)	35 (11)	34 (11)
Ethnic background: Caucasian	35/36	12/12	23/24
BMI (kg/m^2^) (mean and standard deviation)	22.9 (2.9)	24.3 (2.5)	22.2 (2.8)
Oral contraceptive (%)	33	NA	50
Current smoker (%)	22	25	21
Caffeine consumption (cups/day) (mean)	3	3	3
Alcohol consumption (glasses of wine/ day) (mean)	0.08	0.08	0.08

*BMI* body mass index.

Paracetamol concentrations varied between 5 and 500 mcg /mL in urine samples and between 1 and 100 mcg /mL in plasma samples, values in accordance with the reported paracetamol concentrations in biological samples^33,40,41^.

We analysed three different metabolic ratios: PS/PG, PS/(P + PS + PG) and PS/(P + PS + PG + PM + PC). Considering the three ratios, the ratio PS/(P + PS + PG) (hereafter referred to as PSI) resulted in the least amount of intraindividual variability. Although only the results for this ratio are reported, statistical analysis was performed for the three ratios, producing similar results (data not shown).

### PSI distribution and variability

**Figure 2:**
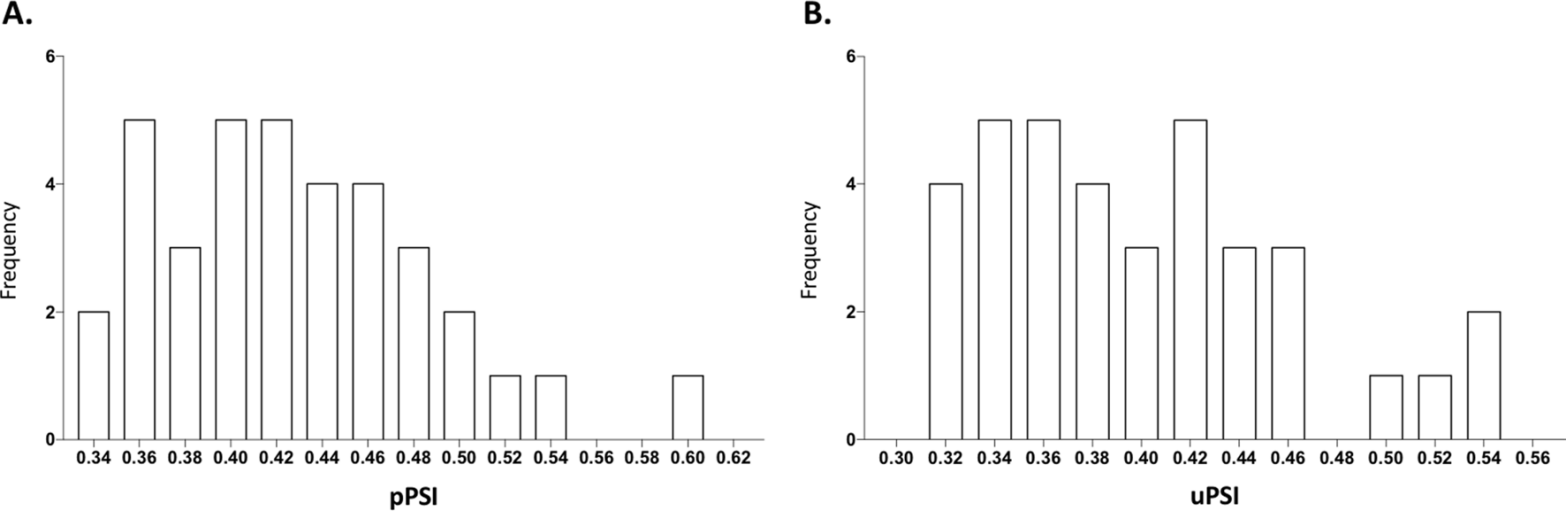
Frequency histogram plotted as Paracetamol Sulfonation Index (PSI) for clinical phenotyping of SULT vs. number of individuals (n = 36). Abscissa denotes the metabolic ratio expressed as PSI, calculated from unchanged paracetamol and its sulfate and glucuronate conjugates, measured in plasma **(A)** and urine **(B)** samples using LC-HRMS, 2 h after ingestion of 1 g of paracetamol. We found a similar distribution of PSI for plasma **(A)** and urine **(B)** samples, revealing an identical mean and a right skew. Although the distribution is mostly uniform, individuals with higher sulfonation capacity may be singled out.

The frequency distribution histogram of plasma PSI (pPSI) and urinary PSI (uPSI) was positively skewed in the studied population (Fig. 2). The mean pPSI and uPSI were 0.43 (range 0.34 to 0.60) and 0.40 (range 0.31 to 0.55) respectively.

#### Intraindividual variability

**Figure 3:**
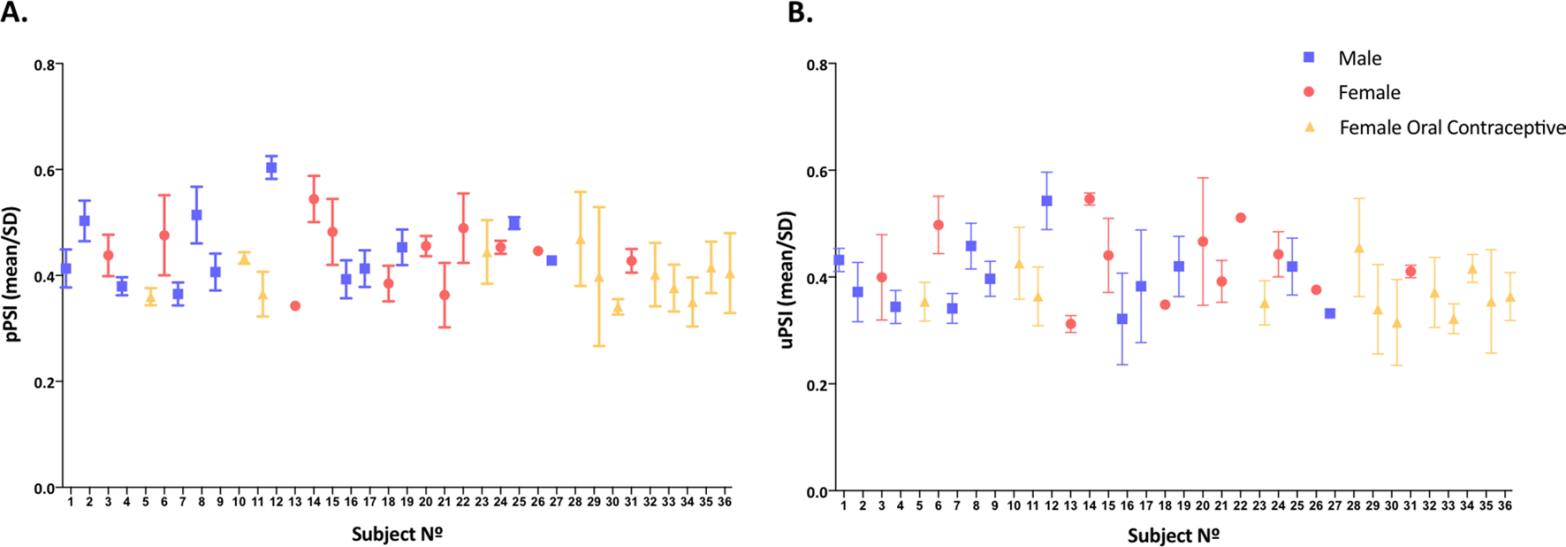
Mean Paracetamol Sulfonation Index (PSI) for clinical phenotyping of SULT in 34 individuals. PSI was calculated from unchanged paracetamol and its sulfate and glucuronate conjugates, measured in plasma **(A)** and urine **(B)** samples using LC-HRMS, after ingestion of 1 g of paracetamol on three separate occasions. Data are mean and standard deviation (SD) calculated for every subject; only one value is presented for individuals No. 26 and No. 27, who missed two phenotyping visits.

Figure 3 illustrates the PSI for 23 women and 11 men who underwent consecutive PSI measuring. Only one value is presented for the female subject and the male subject who had data collected out of phase because they missed their appointments.

No differences were found between men and women. Coefficients of intraindividual variation in PSI ranged from 2 to 33% in plasma and 2% to 28% in urine, with a mean value of 10% and 13%, respectively.

#### Influence of gender

**Figure 4:**
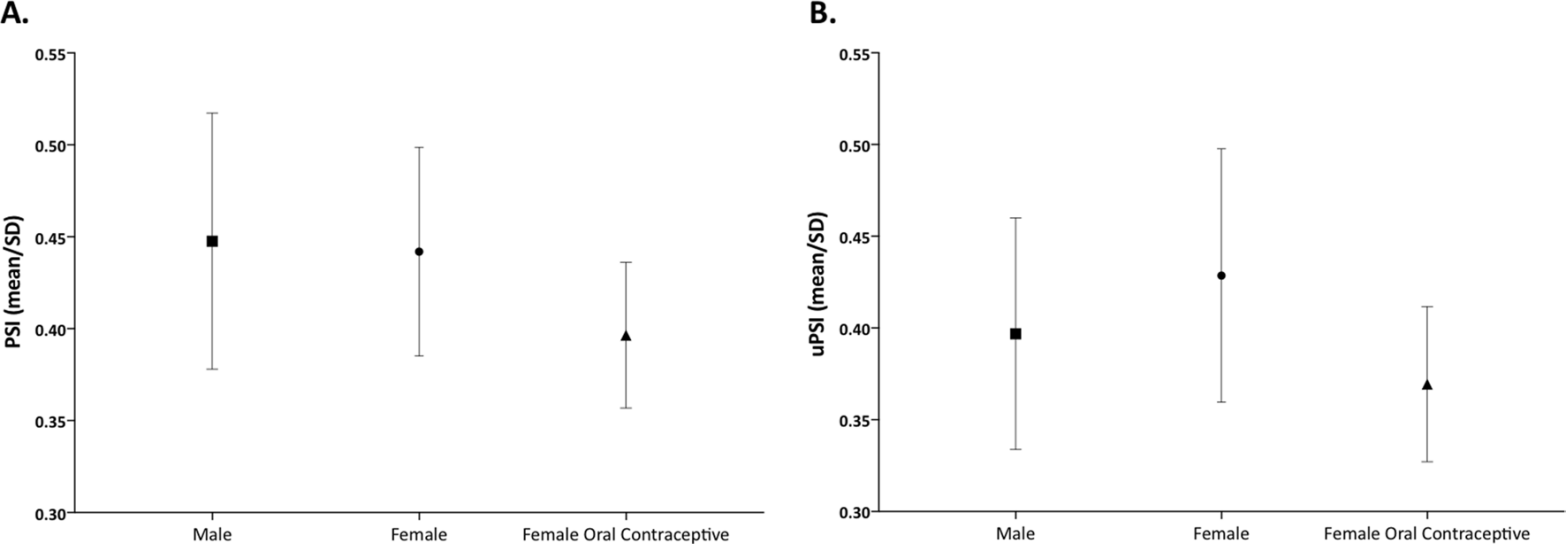
Comparison of Paracetamol Sulfonation Index (PSI) for clinical phenotyping of SULT between men and women, on and off oral contraceptives. Data are mean and standard deviation of PSI calculated in plasma **(A)** and urine **(B)** samples for 12 men, 12 women off oral contraceptives and 12 women on oral contraceptives. No significant differences were found between the three groups.

Figure 4 illustrates the pooled PSI for men and for women, and no sex-dependent disparities were found, both in plasma and urine samples.

#### Influence of oral contraceptives

Figure 4 depicts the pooled values for women on and off oral contraceptives and there were no differences between groups, both in plasma and urine samples.

### Relationship between PSI in urine and renal clearance

In order to investigate whether PSI values in urine were affected by renal clearance, we analysed the influence of glomerular function (estimated glomerular filtration rate—eGFR) estimated through the CKD-EPI 2019 Creatinine Equation^34^ and passive reabsorption measuring urinary pH.

No correlation was found between PSI in urine and urine pH or eGFR.

### Correlations among urinary and plasma measurements

No concordance was found between values of unchanged paracetamol in plasma and urine. However, a statistically significant correlation was found for all the individual metabolites (glucuronide, sulfate, cysteinyl-S conjugates and mercapturate) between plasma and urine. The Pearson’s correlation coefficient measuring the linear association between the two variables was lowest for PS (Pearson r = 0.61) and highest for PC (Pearson r = 0.84).

**Figure 5:**
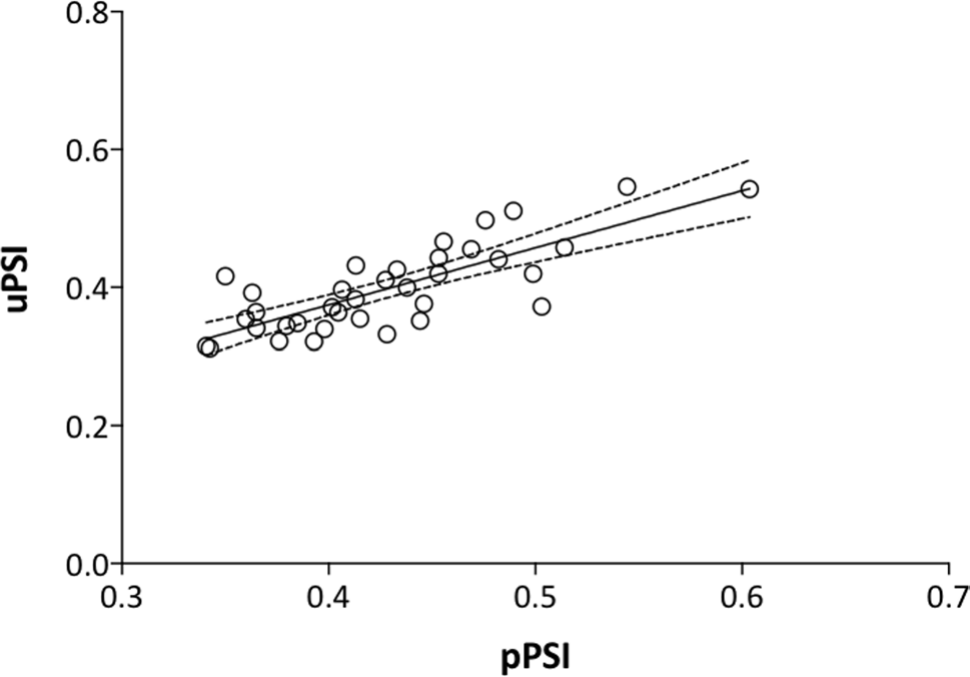
Correlation between Paracetamol Sulfonation Index (PSI) for clinical phenotyping of SULT measured in plasma and urine samples. We found a good correlation between plasma and urinary PSI (Pearson’s correlation coefficient = 0.79, *p* < 0.001).

The correlation between plasma PSI and urinary PSI was also different from zero with statistical significance and the estimated Pearson’s correlation coefficient obtained was 0.79 (Fig. 5).

### Tolerability of probe drug

Paracetamol was well tolerated in all administrations and no adverse events were considered related to the medicinal product.

## Discussion

In this population of healthy adults, PSI showed low intraindividual variability, with a good correlation between values in plasma and spot urine samples. Urinary PSI was independent of factors not related to enzyme activity such as urine pH or eGFR. Gender and oral contraceptive intake had no influence on our metabolic ratio.

So far, studies on variability of SULT activity were carried in animals or in vitro^2^, using enzymatic activity or expression studies in cell cytosolic fractions^14^. The few existing clinical studies estimating sulfonation capacity were focused on differences on sulphur metabolism across disease states, rather than on designing a validated method to measure SULT activity^17–22^.

Compared to these previous studies of SULT activity, our work’s major innovation is the intent to measure sulfoconjugation in clinical context. To do so, we chose a metabolic phenotyping method. Metabolic profiling is now a well-accepted integrative approach to stratify patients according to the activity of drug metabolizing enzymes, sensitive to both genetic and environmental influences, and has been used extensively to study cytochromes^42^. The same is not true among phase 2 enzymes, where N-acetyltransferase type 2 is the only one with an established probe substrate^43^.

### Paracetamol as a probe for the study of sulfonation

Others before us have used paracetamol to study sulfonation but with different purposes. In a seminal work on SULT from the 80’s, Reiter et al. studied SULT enzymatic activity in platelet cytosols and its correlation with in vivo sulfate conjugation of paracetamol, concluding ﻿﻿that variations in platelet SULT activity reflect individual variations in the urinary excretion of the sulfate conjugate of paracetamol^44^. In the same decade, Bonham-Carter used a similar design to test the correlation between SULT activity measured with different substrates and the in vivo conjugation of paracetamol and salicylamide in man, but came to a different conclusion: they found no significant relationship between the in vivo pattern of sulfoconjugation of either drug and the activity of platelet SULT assayed with tyramine^45^. Throughout de 90s, Waring et al. produced vast literature devoted to studying variations in sulphur xenobiochemistry in pregnancy, circadian rhythm and several chronic diseases, including autism, migraine, inflammatory arthritis, biliary cirrhosis and chronic neurological diseases^17–22,46,47^. This team used paracetamol sulfate and the ratio PS/PG as an estimate of sulfation capacity, leading to breakthroughs such as scientific evidence suggestive of abnormal sulfur metabolism affecting people with autism spectrum disorders. Nevertheless, no intention was dedicated to validating the method. In a paper from 2009, Clayton et al., intent on investigating the prediction of drug response based on the predose urinary metabolite profile, chose paracetamol to exemplify this pharmacometabonomic approach; on doing so, they uncovered important interactions between sulfonation of xenobiotics and host microbiome metabolites^48^. In 2019, Cook and co-authors, to demonstrate that SULT isoenzymes could be selectively inhibited for therapeutic purposes, administered both paracetamol and mefenamic acid (a known SULT1A1 inhibitor) to an adult healthy man and found that paracetamol sulfonation was substantially decreased^49^.

Our choice of paracetamol as a probe drug proved to be a very convenient one, fulfilling all the necessary requirements for such a compound^43^: a widely available, safe and inexpensive therapeutic drug; with rapid absorption upon oral administration and a short elimination half-life; and finally with a significant metabolism by SULT at therapeutic doses and a simple metabolic scheme. On the downside, paracetamol is not exclusively metabolized by SULT; nevertheless, it is directly sulfonated without a phase I intermediate and it has a sufficient degree of selectivity to SULT1A1 and SULT1A3. Moreover, as paracetamol is glucuronidated and sulfonated at therapeutic doses and oxidative metabolism is a minor contributor, it is a useful model compound for the study of phase II conjugation^50^. So far, clinical research on metabolic phenotypes has been more focused on CYP450 enzymes and evidence is needed for phase II enzymes.

The main isoforms involved in paracetamol metabolism in adults are SULT1A1 and SULT1A3^10^. Still, we know from previous studies that the production of paracetamol sulfate is not influenced to a significant extent by the route of administration (oral versus intravenous), implying there is little sulfonation in the gastrointestinal tract, supporting a principal role for the major hepatic isoform SULT1A1, rather than the major intestinal isoform SULT1A3, in the metabolism of paracetamol in vivo^51^. While using paracetamol as a probe substrate, we can assume that we are evaluating the SULT enzymes involved in metabolizing orally administered phenolic drugs, predominantly the activity of SULT1A1, the main SULT isoform in drug metabolism.

### PSI as a metric for the study of sulfonation

By administering a fixed dose of paracetamol to subjects, we could measure the concentration of the drug and its metabolites and build a formula to infer the activity of phenol SULT: Paracetamol Sulfonation Index (PSI). This metric offers several desirable qualities. First, the metric appears sensitive enough to discriminate between subjects with higher and lower phenol sulfonation capacity, as we can observe from the frequency histogram.

Secondly, PSI both in plasma and urine showed a low intraindividual coefficient of variation suggesting high individual stability of SULT activity and good reproducibility of our ratio. This is of outstanding importance for a phenotyping metric^52^.

Thirdly, our metric revealed good correlation between values in plasma and urine. So, for further studies we can use the metabolic ratio in urine spot samples due to the non-invasiveness and ease of sampling.

Finally, urinary PSI does not depend on factors not related to enzyme activity such as urinary pH and renal function; this is line with the reports of the renal clearance of paracetamol and ﻿the highly polar glucuronide and sulfate conjugates, rapidly excreted by the kidney without reabsorption, being relatively insensitive to variations in urine flow, urine pH and kidney function^32^.

The accuracy with which our probe drug metric, PSI, indeed reflects the activity of phenol SULT is difficult to scrutinize since there are no other validated metrics for SULT activity in vivo and there is poor correlation between in vitro and in vivo activity^45^. Providentially, the evidence collected by Cook and co-authors in 2019 by co-administering paracetamol and mefenamic acid to an adult male implicitly validates our metric: they proved that a known SULT1A1 inhibitor reduced the generation of paracetamol sulfate, so we can infer PSI is sensitive to enzymatic inhibition.

It was not feasible to relate our metric to genetic polymorphisms, since these are ethnically distributed and are not anticipated in an ethnically homogeneous population such as ours. Furthermore, genetic variation only explains a minor part of interindividual variability in SULT activity^15^.

Thus, as a general caveat, PSI may reflect a number of variable pharmacokinetic processes that influence phenol sulfonation, such as cofactor availability or variation in alternative metabolic pathways like glucuronide conjugation. It will not give us mechanistic information, but it will provide us an empirical measure of an individual’s sulfonator status for phenolic compounds, as previously hinted by RH Waring’s clinical research.

### First insights into interindividual variability of PSI

Differences in sulfonation were not found between men and women. This is in sharp contrast with the documented sexual dimorphism of SULT expression in rodents^53^ and in accordance with previous studies in humans that found no gender-related difference in the expression of various SULT isoforms in the liver^54^ and in SULT1A1 enzymatic activity in liver cytosols^55^. It suggests that stratification need not be done on the basis of gender for pharmacokinetic investigations of drugs that are SULT1A1 substrates. There were also no differences in PSI between women on and off oral contraceptives, suggesting: (1) no influence of menstrual cycle hormonal fluctuations on sulfonation, (2) no relevant inhibition of sulfonation of a single therapeutic dose of paracetamol by oral contraceptives, in contrast with previous in vitro and in vivo results^47,56^. Obviously, these data will have to be confirmed in a larger population sample.

In conclusion, SULT phenotyping using paracetamol as a probe may provide a useful tool for implementing precision medicine initiatives for phenolic drugs mainly metabolized by SULT. To the best of our knowledge, our study constitutes the first attempt to standardise a phenotyping method that provides an in vivo empirical measure of an individual’s sulfonator status. It is a simple non-invasive method requiring urine spot samples, using the safe and convenient paracetamol as a probe drug, and with low intraindividual coefficient of variation.

To gain further knowledge on PSI, we are now studying a population of patients with chronic disease, with different comorbidities and environmental exposures, and under different drugs. We expect that analysing the interindividual variation in this population will provide new insights into our metric as, among other observations, we record variation in PSI for patients treated with known inhibitors/inducers of the enzyme or in relation to liver and other states of disease.

## Supplementary Information

Supplementary Tables.

## Data Availability

The datasets generated and analysed during the current study are available from the corresponding author on reasonable request.
