# Supplementary material for: A simple method to measure sulfonation in man using paracetamol as probe drug
Source: Sci Rep. 2021 Apr 27;11:9036. doi: 10.1038/s41598-021-88393-3 (PMC8079418; doi:10.1038/s41598-021-88393-3)
Supplement: Supplementary file 1 — Supplementary Tables. [file 41598_2021_88393_MOESM1_ESM.docx]

**SUPPLEMENTARY INFORMATIONS**

**Title:** Clinical phenotyping of SULT in man: a simple metric using paracetamol as probe

**Authors:**

Natália Marto, Judit Morello, Alexandra M M Antunes, Sofia Azeredo, Emília C Monteiro, Sofia A Pereira

**Supplementary Table S1. Assignment of MS and MS/MS spectra of paracetamol and paracetamol metabolites in a pool of urine samples.** Precursor ions are shown in bold. Only, those ions with an intensity > 10,000 units are shown in the table.

|  | **Retention**  **time (min)** | **Measured accurate mass** | **Ion Formula** | **Exact**  **mass** | ***∆* (mDa)** | **mSigma** |
| --- | --- | --- | --- | --- | --- | --- |
| **Paracetamol** | 3.0 | **152.0711** | **C_8_H_10_NO_2_** | **152.0706** | **0.5** | **8.7** |
|  |  | 110.0688 | C_6_H_8_NO | 110.0600 | 8.8 | 9.8 |
| **Paracetamol glucuronide** | 2.7 | **328.1031** | **C_14_H_18_NO_8_** | **328.1027** | **0.4** | **26.7** |
|  |  | 152.0708 | C_8_H_10_NO_2_ | 152.0706 | 0.2 | 9.5 |
| **Paracetamol sulfate** | 2.8 | **232.0278** | **C_8_H_10_NO_5_S** | **232.0274** | **0.4** | **6.9** |
|  |  | 152.0708 | C_8_H_10_NO_2_ | 152.0706 | 0.2 | 7.7 |
| **Paracetamol cysteine** | 2.8 | **271.0755** | **C_11_H_15_N_2_O_4_S** | **271.0747** | **0.8** | **3.0** |
|  |  | 208.0425 | C_10_H_10_NO_2_S | 208.0429 | -0.4 | 11.9 |
|  |  | 182.0268 | C_8_H_8_NO_2_S | 182.0270 | -0.2 | 10.4 |
|  |  | 140.0169 | C_6_H_6_NOS | 140.0165 | 0.4 | 14.3 |
| **Paracetamol mercapturate** | 3.4 | 313.0859 | **C_13_H_17_N_2_O_5_S** | **313.0853** | **0.6** | 9.9 |
|  |  | 295.0756 | C13H15N2O4S | 295.0747 | 0.9 | 13.6 |
|  |  | 271.0751 | C11H15N2O4S | 271.0747 | 0.4 | 5.7 |
|  |  | 254.0482 | C11H12NO4S | 254.0482 | 0 | 11.4 |
|  |  | 225.0693 | C10H13N2O2S | 225.0692 | 0.1 | 10.6 |
|  |  | 208.0425 | C_10_H_10_NO_2_S | 208.0427 | -0.2 | 9.2 |
|  |  | 182.0269 | C_8_H_8_NO_2_S | 182.0270 | -0.1 | 17.2 |
|  |  | 166.0320 | C_8_H_8_NOS | 166.0321 | -0.1 | 10.6 |
|  |  | 140.0174 | C_6_H_6_NOS | 140.0165 | 0.9 | 8.1 |

**Supplementary Table S2. Assignment of MS and MS/MS spectra of paracetamol and paracetamol metabolites in a pool of plasma samples.** Precursor ions are shown in bold. Precursor ions are shown in bold. Only, those ions with an intensity > 10,000 units are shown in the table.

|  | **Retention**  **time (min)** | **Measured accurate mass** | **Ion Formula** | **Exact**  **mass** | ***∆* (mDa)** | **mSigma** |
| --- | --- | --- | --- | --- | --- | --- |
| **Paracetamol** | 2.9 | **152.0710** | **C_8_H_10_NO_2_** | **152.0706** | **0.4** | **5.2** |
| **Paracetamol glucuronide** | 2.7 | **328.1032** | **C_14_H_18_NO_8_** | **328.1027** | **0.5** | **9.4** |
|  |  | 152.0711 | C_8_H_10_NO_2_ | 152.0706 | 0.5 | 20.4 |
| **Paracetamol sulfate** | 2.7 | **232.0275** | **C_8_H_10_NO_5_S** | **232.0274** | **0.1** | **8.2** |
| **Paracetamol cysteine** | 2.8 | **271.0752** | **C_11_H_15_N_2_O_4_S** | **271.0747** | **0.5** | **11.7** |
| **Paracetamol mercapturate** | 3.3 | **313.0855** | **C_13_H_17_N_2_O_5_S** | **313.0853** | **0.2** | **42** |
